# Employees’ attitudes and work-related stress in the digital workplace: an empirical investigation

**DOI:** 10.3389/fpsyg.2025.1546832

**Published:** 2025-02-11

**Authors:** Maddalena Cavicchioli, Fabio Demaria, Francesca Nannetti, Anna Chiara Scapolan, Tommaso Fabbri

**Affiliations:** ^1^Marco Biagi Department of Economics, University of Modena and Reggio Emilia, Modena, Italy; ^2^Department of Communication and Economics, University of Modena and Reggio Emilia, Reggio Emilia, Italy

**Keywords:** work-related stress, digital workplace, employees’ attitudes, digitalization, virtual meetings, job satisfaction, work-life interface, person-organization fit

## Abstract

**Introduction:**

In the digital era, the integration of advanced, hyper-connected technologies deeply reshaped work dynamics and organizational practices, especially through the transformation of the spatial and temporal dimensions of work. This study examines the complex relationship between digitalization and work-related stress, with a particular emphasis on the impact of both digital activities (i.e., number of actions and virtual meetings) and employees’ attitudes (i.e., job satisfaction, person-organization fit, engagement, and work-life interface) on stress levels.

**Methods:**

Drawing on data from Microsoft 365 activity records and an online survey of employees in an Italian AI firm, this study applies Factor Analysis and Generalized Additive Models to analyze the above-mentioned interactions in a highly digitalized context.

**Results:**

Findings indicate that the time–space intensification and extension of the working experience has a significant impact in terms of increasing work-related stress when exceeding certain number of off-hours digital actions and virtual meetings. Conversely, job satisfaction, perceptions of work-life enrichment and person-organization fit represent subjective employees’ attitudes that significantly influence and reduce work-related stress in a digitalized working environment.

**Discussion:**

This study contributes to the existing academic literature by providing a more nuanced understanding of the dual impact of work digitalization on employees’ well-being. Furthermore, our findings offer practical insights into the management of employees and their level of stress in digital work settings.

## 1 Introduction

The pervasiveness of the increased use of digital technologies is commonly associated with crucial changes within organizations (Hanelt et al., 2021; Schwarzmüller et al., 2018). More precisely, the introduction and massive employment of the most advanced and sophisticated Information and Communication Technologies (ICTs) in working environments requires attention to be focused on the interaction between people and technology within the organization (Imran et al., 2021), a complex system that consists of interdependent components (Davis et al., 2014), both tangible and intangible, structurally embedded within everyday practices. Indeed, drawing on the existing literature, there is consensus that the inherent complexity of this phenomenon involves a deep and systematic reshaping of working processes, encompassing strategies, structures, culture, and leadership (Hess et al., 2016; Kane et al., 2015; Nadkarni and Prügl, 2020).

However, assuming the perspective that digitalization is not simply about technology but requires a specific focus on employees’ factors (Trenerry et al., 2021), much of the literature on this never stopping phenomenon can be readily divided into optimistic and pessimistic accounts, given the amount of both foreseen and unforeseen risks and opportunities that this transformation brings with it (Amankwah-Amoah et al., 2021). Indeed, as noted by Shah et al. (2017, p. 366), “the dominant focus of change and how it is managed within organizations remains at the level of employees’ engagement—who, in terms of adopting change may develop positive or negative attitudes, beliefs and intentions towards the organization as change is implemented.”

This suggests that further investigation into how organizations can effectively manage digitalization should take into consideration both the digital working experience and the attitudes of employees, with the aim of highlighting significant relationships between these factors and work-related stress in the context of technology integration into daily work activities. It is evident that the use of ICTs has the potential to shape work dynamics in ways that can both support and challenge employees’ well-being (Chesley, 2014). On the one hand, the proliferation of digital technologies enables greater flexibility in the organization of work activities (Hokke et al., 2024), facilitates knowledge sharing and creativity, fosters interactions with colleagues and supervisors (Bolli and Pusterla, 2022; Castellacci and Viñas-Bardolet, 2019) and enhances individual autonomy over time and location of work (Vega et al., 2015), promoting satisfaction and work-life balance (Alieva and Powell, 2022). On the other hand, the intensification of the pace of work while working due to digital scheduling (Berg et al., 2023) and the extension of office hours beyond traditional boundaries as a consequence of the ubiquity of technology (Beer and Mulder, 2020; La Torre et al., 2019;) can lead to detrimental effects on physical and mental well-being, generating for instance work-life conflict (Stich et al., 2018). In this regard, among the different types of work-related stress, technostress (Brod, 1984) assumes a particularly relevant position in the contemporary workplace. This form of stress arises in technology-driven workplaces, where employees face challenges such as constant connectivity, frequent interruptions, and the overwhelming flow of information. Therefore, the existence of divergent perspectives within the existing literature pertaining to the impact of digitalization on employee wellbeing calls for further empirical research that specifically addresses work-related stress experienced in a digitalized work setting. Consequently, this article aims to provide useful insights specifically exploring what sort of influence the time–space intensification and extension of working experience (in terms of number of digital tasks and online meetings) and employees’ attitudes towards their job and organization (including job satisfaction, person-organization fit, engagement, work-life interface) have on work-related stress within a deeply digitalized workplace. To effectively address this research question, an online self-administrated survey has been submitted to 144 employees operating in a highly digitalized Italian firm specialized in Artificial Intelligence (AI) solutions, and data from the Microsoft 365 platform was collected.

Our empirical analysis confirms previous research on technostress induced by digitalization, by demonstrating that the dissolved spatial and temporal boundaries of work— evidenced by constant video conferencing and out-of-hours work—significantly increase employees’ stress levels. Specifically, our findings indicate that performing around 250 work-related actions outside office hours in a single day, as well as attending more than 11 meetings per week on average, leads to a notable rise in work-related stress. We thus refined existing studies, identifying specific thresholds of work intensification and extension that contribute to technostress.

However, our analysis also reveals that job satisfaction, effective management of the work-life interface (i.e., work-life enrichment), and perceived organizational fit play a crucial role in reducing employees’ stress levels. Thus, this study contributes to the existing academic literature by providing a more nuanced understanding of the dual impact of work digitalization on employees’ well-being. Specifically, our key contribution lies in highlighting how employees’ attitudes toward their job and organization can act as mitigating factors against the stress stemming from the intensified and extended work time–space in highly digitalized environments. Drawing upon these findings, we offer practical insights into the management of employees and their level of stress in digital work settings.

This paper is structured as follows. The next section examines the existing literature on digital transformation and its impact on employees’ experience of their work, followed by the presentation of the hypotheses that have been formulated. Section 3 provides a detailed explanation of the empirical research, which is based on a Confirmatory Factor Analysis (CFA) and Generalized Additive Models whose main results are presented in Section 4. Finally, Section 5 presents a discussion of the findings, identifies practical contributions, current limitations and areas for further research.

## 2 Theoretical framework and hypotheses

As mentioned above, recent decades have seen radical changes in how people live and work, due to the pervasiveness of the online realm. Consequently, in the digitalization era, to understand how people respond to their working experience it is necessary to consider that digital and hyper-connected technologies transformed the spatial, as well as the temporal dimension of work, largely enabling new ways of working (Corvello et al., 2022). According to Giacosa et al. (2023), ICTs have disrupted the foundations of organizational culture and revolutionized the daily work routine of most companies, forcing them to reflect on how to adapt their activities and internal processes.

Moreover, Fabbri et al. (2019) argue that the increased adoption of collaborative platforms and digital workplaces by companies to facilitate work, communication, and collaboration, with the additional objective of fostering innovation and promoting innovative work practices, inevitably affect the way people feel and behave. A considerable amount of literature has been published on the impact of digitalization on individuals’ working experience, since, as suggested by Giacosa et al. (2023), some innovations that are here to stay, such as teleworking, deeply characterize the post-pandemic workplace. These studies can be divided into those that highlight the dark side of technology (Bondanini et al., 2020), emphasizing the negative effects of its introduction at work (Berg-Beckhoff et al., 2017; Burman and Goswami, 2018), and those that instead point out the benefits of ICTs on employees’ working conditions (Ninaus et al., 2021; Vega et al., 2015).

More specifically, the extant literature focused on digital transformation’s benefits has claimed that the advancement of digital technologies provides workers with greater flexibility in work schedules, extensive knowledge sharing across organizational boundaries and control with respect to the time and place of their work (Coenen and Kok, 2014; Ter Hoeven and Van Zoonen, 2015; Vega et al., 2015). According to Cortellazzo et al. (2019), digital technology has influenced organizational dynamics and personnel management by facilitating the utilization of virtual teams. This new organizational structure has positively affected employees’ working experience, reducing travel costs and times (Bergiel et al., 2008) and enhancing innovation and creativity (Gupta and Pathak, 2018). This is consistent with what emerged from the online survey conducted in 2023 by Soffia et al. (2024). Indeed, the results, collected after the forced use of digital technologies imposed by the lockdown pandemic period, suggested that quality of life appeared to be positively correlated with the increased frequency of interaction with digital workplace ICTs. Beer and Mulder (2020, p.13) also noted that employees “could benefit from experimenting and monitoring one’s own strategies for time and attention management.” Moreover, it has also been argued that the rise in connectivity and information sharing is fostering the dissolution of hierarchies and functional and organizational boundaries. This evolution is gradually shifting activities from task-based to more project-based endeavors, necessitating employees’ direct involvement in generating new value-added outcomes (Cortellazzo et al., 2019).

It is widely agreed that in this fast-paced and ever-changing environment, one of the most striking shifts has been that office hours have extended beyond traditional boundaries, which has made work both more dynamic and challenging for employees (Mahboob and Khan, 2016). However, keeping up with the rapid technological advancement of the last few years has also been considered as a stress factor (Shepherd, 2008).

The phenomenon of work-related stress has been extensively investigated and the stress experienced by different occupation types and job roles has been analyzed in many studies (Johnson et al., 2005). Mucci et al. (2015, p. 673) summarize the work-related stress concept as follows: “the product of the dynamic interaction between the person and the social and organizational context in which he or she works, constituting the result of a (not equal) relationship between the stresses imposed by the task/role and the operator’s ability to cope with these.” Of all the different types of work-related stress, technostress inevitably is the most widely discussed in the current era. Brod (1984) first defined technostress as an ineffective coping with technology, due to a combination of performance anxiety, information overload, role conflicts and organizational actors. According to Tarafdar et al. (2007), this concept refers to any adverse effects on attitudes, thoughts, behaviors, or physiological well-being, whether originating directly or indirectly from technology.

In this regard, many studies have pointed out that workers face new types of work-related stress within environments where communication and interaction are dependent on ICTs (Suh and Lee, 2017), having to deal with the perception of information overload and constant availability (La Torre et al., 2019) and to cope with frequent tight deadlines and interruptions deriving from electronic workflows (Agypt and Rubin, 2012; Fonner and Roloff, 2012). According to Beer and Mulder (2020, p. 15) “workload and workflow interruptions increase as a general consequence of the ubiquity of technology, mainly due to a higher level of job speed and the associated time and workload pressure.” Similarly, the research of Chesley’s (2014) reveals that employees who daily use ICTs often perceive their work as requiring rapidity and manifest greater feelings of overwhelm compared to their counterparts who use these technologies less frequently. The time and energy (Stich et al., 2018) expended in managing such interruptions can lead to detrimental effects on productivity (Addas and Pinsonneault, 2015) as well as on physical and mental well-being (Barber and Santuzzi, 2015). For instance, the colloquially called Zoom fatigue (Fosslien and Duffy, 2020) or videoconferencing fatigue “refers to the extent to which people experience exhaustion that is directly linked to their participation in videoconferences” (Luebstorf et al., 2023, p.151). For example, Abramova and Gladkaya (2024), whose first study was conducted shortly after the first COVID-19 outbreak, while the second one was completed a year later, confirmed the criticality of videoconferences’ duration as a predictor of exhaustion and found that “the effects of self-view frequency are significant for negative affect and exhaustion after a VC” (2024, p. 15).

As the existing literature primarily insists on how digital technologies support new ways of working that promote an extension of work “into virtually any space where a smartphone, tablet or laptop can be operated” (Hassard and Morris, 2022, p. 1651), with digital scheduling intensifying the pace of work while working (Berg et al., 2023) and multitasking requiring to switch “back and forth between different work tasks in a relatively short time” (Luebstorf et al., 2023, p. 159), we first investigated the relationship between digital time–space intensification and extension of working experience and work-related stress. Therefore, we formulated our first hypothesis as follows:

*H1*: In a digitalized working context, time–space intensification and extension of working experience significantly increases work-related stress.

However, from all the above-mentioned studies, it appears as not clear whether and how possible negative effects (such as, for instance, work-related stress) may interact with potential positive impacts of work digitalization (in terms of positive employees’ attitudes towards job and organization, such as work-life balance, engagement or satisfaction). Therefore, we addressed this issue by formulating a set of research hypotheses on the potential relationships that work digitalization may feed between employees’ attitudes and work-related stress.

### 2.1 Digitalization, work-life interface, and work-related stress

The digital devices’ invasion of private life, the possibility to constantly monitor and immediately respond to work-related messages (Richardson, 2017), but also the perception to be always available for work, the necessity to cope with multi-tasking (De et al., 2020) and the greater working time flexibility under workers’ control (Berg et al., 2023) are some of the effects of digital transformation on employees’ work-life interface. Work and family are two broad domains of an individual’s life that clearly interact and have influence on each other: how people react to and cope with this interaction largely affect both the individual himself and the organization (Carlson and Kacmar, 2000). In the intricate interface between work and personal life, while work and family responsibilities may interfere with one another, research indicates that resources acquired in one domain can also improve the quality of life in the other (Greenhaus and Powell, 2006; Siu et al., 2010). Accordingly, the positive side of the work-life interface is known with the expression of work-life enrichment, which refers to the way in which work and family benefit each other (Carlson et al., 2006). The other side of the work-life interface is precisely referred to as work-life conflict, “a form of interrole conflict in which the role pressures from the work and family domains are mutually incompatible in some respect. That is, participation in the work (family) role is made more difficult by virtue of participation in the family (work) role” (Greenhaus and Beutell, 1985, p. 77).

A consensus exists on the evidence that work-life conflict produces stress-related outcomes (Anderson et al., 2002; Fiksenbaum, 2014), representing one of the prevalent sources of work-related stress for both men and women (Frone, 2000). At the same time, the extant literature suggests that work-life enrichment positively relates with individual’s mental health (Baral and Bhargava, 2010).

As mentioned before, ICTs blurred (Cijan et al., 2019) boundaries between job and personal life, transforming the spatial and the temporal dimension of both work and private life. As reported in Chesley (2014), likewise ICTs usage can enable the extension of work into personal time, similar ICT-driven practices can also allow non-work-related concerns or demands to infiltrate the workplace. Therefore, the flexibility guaranteed from the use of digital technologies is strictly related to the dimension of work-life interface. This new work dynamic can have positive effects on the interaction between the professional and intimate spheres (Ratna and Kaur, 2016; Towers et al., 2006;) if enabling not only a better balance between them (Alieva and Powell, 2022) but also improving an “enrichment.”

Indeed, Hokke et al. (2024) found that employees who benefit from flexible work arrangements tend to report lower levels of work-to-family conflict, increased enrichment, and greater satisfaction with work-life balance compared to those without such flexibility.

On the other hand, such constant availability facilitated by ICTs has also been linked to heightened conflict between work and personal life (Derks et al., 2015; Stich et al., 2018), causing negative effects (Derks et al., 2014; Towers et al., 2006), colonizing home-life (Berg et al., 2023) and making it very difficult to completely switch off from work and recuperate (Grant et al., 2013). As stated by Marsh et al. (2024), whose study is framed in a “context of widespread remote and hybrid working practices, especially post-pandemic,” hyper-connectivity and overload can have detrimental effects on both the physical and mental wellbeing of employees.

Accordingly, we hypothesized that in a digitalized workplace, employees’ work-life interface significantly relates to work-related stress and specifically we formulated the second hypothesis as follows:

*H2a*: In a digitalized working context, employees’ work-life enrichment significantly reduces work-related stress.

*H2b*: In a digitalized working context, employees’ work-life conflict significantly increases work-related stress.

### 2.2 Digitalization, positive employees’ attitudes towards their job and organization, and work-related stress

Despite the evidence that technological innovation influences work intensification processes and employees’ levels of work-related stress, Chesley (2014, p. 607) argues that “the image of the ‘technologically tethered’ worker or the fragmented, stressful workplace is too limited,” because ICTs use has the capability to influence work dynamics that are both problematic and helpful for employees’ well-being. For instance, even though some research conducted during the Covid-19 pandemic observed that since the onset of remote work, there has been a decline in work engagement (Syrek et al., 2022), other studies revealed that home as a work environment facilitates employees’ engagement (Mäkikangas et al., 2022) and remote work contributes to higher levels of work engagement. More recently, Hooi and Chan (2023) affirmed that workplace digitalization positively influences employees’ engagement, enhancing resources for achieving work-related goals. Similarly, Chan et al. (2021) investigated the role of digital literacies in workers engagement in a digitalized workplace, suggesting that workplace digitalization positively influences workers engagement at a significant level and enhancing digital literacy enables employees to remain actively engaged while embracing digitalization in the workplace.

Additionally, it has also been found that occupational stress translates into lower engagement (Anthony-McMann et al., 2017) and when work engagement increases, stress tends to decrease, and performance tends to increase (Junça Silva and Lopes, 2023). Therefore, we formulated the following third research hypothesis to examine the relationship between employees’ engagement and work-related stress in a digitalized workplace:

*H3a*: In a digitalized working context, employees’ engagement towards organization significantly reduces work-related stress.

*H3b*: In a digitalized working context, employees’ engagement towards organization significantly increases work-related stress.

Furthermore, it has been suggested that the mechanisms by which digitalization positively affects workers’ experience have implications for job satisfaction (Soffia et al., 2024). Specifically, most empirical studies focusing on the wellbeing implications of technological transformations of work have found that digitalization has a positive influence on job satisfaction (Alieva and Powell, 2022) since it facilitates information and communication access and sharing (Castellacci and Viñas-Bardolet, 2019; Martin and Omrani, 2015). Bolli and Pusterla (2022, p. 264) explored different pathways through which digital technologies have a potential impact on job satisfaction and suggested that “digitalization affects job satisfaction by first changing some characteristic of the job itself, and then that change impacts the worker’s job satisfaction. Therefore, all of the channels through which digitalization might affect job satisfaction are changes in job characteristics caused by digitalization.” Specifically, they found that the association between digitalization and job satisfaction is positive on average, basically by enhancing work productivity, making work more engaging and fostering interactions with colleagues and supervisors.

Furthermore, a considerable amount of literature reported that job satisfaction strongly links with stress (Stamps and Piedmonte, 1986), suggesting that that there is a significant negative relationship between these interrelated attitudes (Ahsan et al., 2009; Klassen et al., 2010) so that low job satisfaction is associated with high stress (Brown et al., 2012), while the effects of stress can be alleviated by high levels of job satisfaction (Fletcher and Payne, 1980).

Drawing on this evidence, we formulated our fourth research hypothesis as follows:

*H4*: In a digitalized working context, employees’ job satisfaction significantly reduces work-related stress.

Finally, since digitalization is a process of organizational change and cultural transition that involves the internalization of new values and beliefs (Leal-Rodríguez et al., 2023), we concentrate on person-organization fit (P-O fit), defined as “compatibility between people and organizations that occurs when: (a) at least one entity provides what the other needs, or (b) they share similar fundamental characteristics, or (c) both” (Kristof, 1996, p. 4–5).

It is acknowledged that P-O fit contributes to the employees’ embeddedness in the organization, positively affecting task performance, extra-role behaviors and intention to stay (Lee et al., 2014; Mitchell et al., 2001). Furthermore, when P-O fit is high, workers are better equipped to comprehend and effectively implement the organization’s core requirements for innovation (Zhao et al., 2021). Thus, as suggested by Leal-Rodríguez et al. (2023, p. 12), the alignment between people and organization’s culture and the willingness and commitment of employees to understand and embrace digitalization significantly impact the performance, too. Kristof (1996, p. 28) also suggested that when individuals join organizations that match with their characteristics, they experience lower levels of stress than do their “mis-matched” counterparts.

However, job stress usually increases when there is a lack of congruence between employees and organizational characteristics (Mostafa, 2016). Indeed, it has been suggested that the deeper an employee is integrated into the organizational framework, the greater the resources he or she is likely to have accumulated to deal with potential work pressures, including stressful situations and heavy workloads (Chen et al., 2016).

Therefore, we focused on how P-O fit may contribute to the positive effects of the digitalization of work or mitigate its negative impacts on individuals, thereby supporting better employees’ work (and life) experiences, and we formulated the fifth research hypothesis as follows:

*H5*: In a digitalized working context, employees’ perceptions of P-O fit significantly reduce work-related stress.

**Figure 1:**
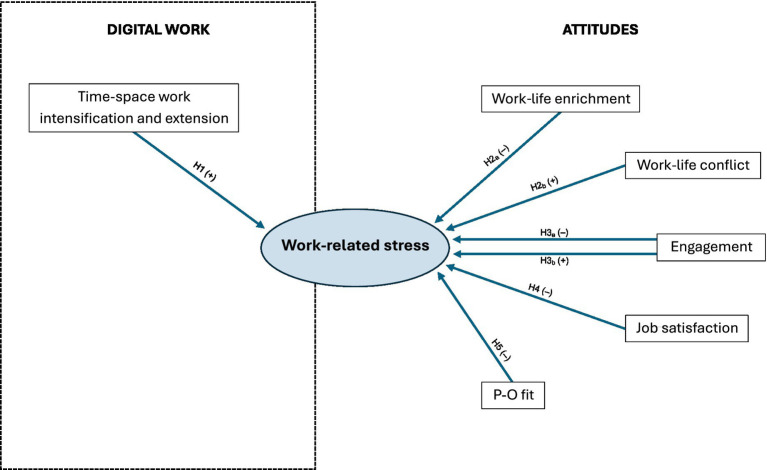
Research hypotheses.

For a summary of all our research hypotheses, see Figure 1.

## 3 Materials and methods

### 3.1 Sample and data collection

The sampling frame for this study encompasses the entire organizational workforce (n = 144) of a consulting company based in Northern Italy. This company actively participated in the research project as an organizational partner through a formal agreement established with the research group. The company specialized in AI solutions across various sectors, including marketing, logistics, and energy, and operates in a fully digital work environment where nearly all activities are conducted via the Microsoft365 platform.

The demographic composition of the sample reveals a significant male predominance, with men representing 75.95% of the workforce. The employees are predominantly young, with a mean age of 34 years, and the majority (62.03%) falling within the 26–35 age range. In terms of education, 91.14% of the workforce holds a university degree, underscoring a highly educated employees’ base. The employment structure is primarily full-time, encompassing 83.54% of the workforce, with the remaining employees engaged in various other contract types. Job roles are distributed across leadership and technical positions, with 39.24% occupying senior roles (including directors and managers) and 60.76% serving as specialists. The average tenure within the organization is 2.7 years, indicating a relatively new but highly skilled workforce. A comprehensive summary of the sample characteristics is presented in Table 1.

**Table 1:** Sample of the study.

Respondents (*n* = 144)	
	Count (%)
Gender	
Female	35 (24.31)
Male	109 (75.69)
Age	
18–25	4 (2.78)
26–35	89 (61.81)
36–45	36 (25)
> 45	15 (10.42)
Education	
Secondary education	13 (9.03)
University degree	131 (90.97)
Role	13 (2.9)
Specialist	87 (60.42)
Manager	27 (18.75)
Head	29 (20.14)
Contract	
Full time	120 (83.33)
Other	24 (16.67)

Data collection was conducted over an eight-month period, from October 2021 to May 2022, and involved the collection of two distinct types of data: survey data and click metadata. In May 2022, six employees’ attitudes were measured using an online self-administered questionnaire distributed via the Microsoft Forms tool on the Microsoft 365 platform of the organization. The survey employed a 7-point Likert scale, with responses ranging from 1 (strongly disagree) to 7 (strongly agree). The items included in the survey and measuring each employees’ attitude were either adopted or adapted from different well-established scales in the existing literature (Goldberg and Williams, 1988; Grzywacz and Marks, 2000; Netemeyer et al., 1996; Ng and Feldman, 2009; Schaufeli et al., 2006; Schneider et al., 2003). To ensure clarity and accuracy in responses, the final questionnaire was translated and adapted into Italian. The decision to use a web-based self-report questionnaire was driven by several advantages, including ease of administration, time efficiency, and the provision of anonymity. Moreover, previous research has shown that online questionnaires can encourage respondents to be more open and truthful in their responses (Meade and Craig, 2012). Participation in the survey was voluntary; however, due to the direct involvement of the organization, the participation rate reached the whole organizational workforce.

The self-administered survey facilitated the collection of primary data, which served as the main variables of interest in this study along with data from the Microsoft 365 platform. This platform generates time-stamped logs documenting every activity undertaken by employees, thereby providing a comprehensive digital record of their work activities. Specifically, during the aforementioned period of inquiry, Microsoft 365 click metadata was sourced and anonymized. Then, we used these clicking behaviors as representative of the time–space intensification and extension of working experience. Lastly, we also collected and employed employees’ demographic data as control variables.

The following section offers a more detailed description of the survey items and control variables used in this study.

### 3.2 Measures

The primary variables of this study are derived from the survey and the Microsoft 365 platform data and can be categorized into dependent, independent, and control variables.

#### 3.2.1 Dependent variable

Work-related stress: this variable is operationalized through three items adapted from Goldberg and Williams (1988). Specifically, these items evaluate respondents’ perceived ability to make decisions, concentrate on tasks, and feel useful within their work environment. An example item include: *“Were you able to concentrate on whatever you were doing?”*

#### 3.2.2 Independent variables

Time–space intensification and extension of working experience: according to Alaimo and Kallinikos (2021) and Fabbri et al. (2022), we operationalized this variable using the following click metadata directly exported from the Microsoft 366 platform’s dashboard: the maximum number of actions taken outside standard working hours (mean = 50.85, SD = 53.208), and the weekly total number of meetings attended (mean = 7.15, SD = 5.59).

Work-Life Enrichment (3 items): This construct assesses the positive effects of work on personal life, such as helping with personal issues, enhancing one’s personal life, and applying job skills in a home setting, as defined by Grzywacz and Marks (2000). For instance, one item states: *“The things I did at work helped me deal with personal and practical issues at home.”*

Work-Life Conflict (3 items): This construct measures the degree of conflict between work demands and family responsibilities, focusing on how work negatively impacts personal life and the strain it causes, as measured by Netemeyer et al. (1996). An example of this measurement is: *“My job produced strain that made it difficult to fulfil family duties.”*

Engagement (6 items): It refers to a positive work-focused psychological state (Halbesleben and Wheeler, 2008; Lesener et al., 2020). Following Schaufeli et al. (2006), engagement was assessed across three dimensions: vigor, absorption, and dedication. Vigor refers to the feeling of energy and resilience at work; absorption reflects a state where time passes quickly due to deep involvement in work tasks; and dedication relates to experiencing a sense of meaningfulness, purpose, and enthusiasm in work. Examples of items measuring engagement include: *“At my work, I felt bursting with energy,” “I felt happy when I was working intensely”* and *“I was enthusiastic about my job.”*

Job Satisfaction (5 items): This construct stands as a pleasurable or positive emotional state that involves a person’s overall evaluation of job, job experiences and job environment (Alegre et al., 2016; Aziri, 2011; Locke, 1976). It captures multiple aspects of job satisfaction, including satisfaction with empowerment, job fulfilment, workgroup dynamics, pay, and job security, as conceptualized by Schneider et al. (2003). One illustrative item in this category is: *“How satisfied are you with your involvement in the decisions that affect your work?”*

P-O fit (3 items): This concept is grounded in the belief that individual attitudes, behaviors, and other outcomes are not solely determined by either the individual or the work environment alone, but rather by the interplay between the two entities (Westerman and Vanka, 2005).Thus, P-O fit measures the extent to which respondents perceive they fit within the company culture, match the organization’s characteristics, and align with its values. Using the scale developed by Ng and Feldman (2009), P-O fit is assessed through items like: *“My values are compatible with the company values.”*

#### 3.2.3 Control variables

In addition to the primary variables, the study incorporates four demographic data as control variables, i.e., gender, age, type of contract, and organizational seniority. These variables are consistent with prior research on digital work and employee attitudes (Bolli and Pusterla, 2022; Chesley, 2014; Ninaus et al., 2021; Olsen et al., 2023; Suh and Lee, 2017). The demographic data were provided by the company and linked to survey respondents via anonymous IDs.

A comprehensive description of all study variables is presented in Table 2.

**Table 2:** Study variables description.

Type of variable	Group	Variable name	Variable description
Dependent	Work-related stress (Goldberg and Williams, 1988)	Stress1	Did you feel capable of making decisions about the things that happened to you?
		Stress2	Were you able to concentrate on whatever you were doing?
		Stress3	Did you feel like you were playing a useful part in the things that happened to you?
Independent	Click metadata	Off-hours actions	Maximum number of actions taken by the user in a working day outside working hours.
		Weekly meetings	Weekly total number of meetings the user attended.
	Work-life enrichment (Grzywacz and Marks, 2000)	Enrich1	The things I did at work helped me deal with personal and practical issues at home.
		Enrich2	The things I did at work made me a more interesting person at home.
		Enrich3	The skills I used on my job were useful for things I had to do at home.
	Work-life conflict (Netemeyer et al., 1996)	Conflict1	Things I wanted to do at home did not get done because of the demands my job put on me.
		Conflict2	My job produced strain that made it difficult to fulfil family duties.
		Conflict3	The amount of time my job took up made it difficult to fulfil family responsibilities.
	Engagement (Schaufeli et al., 2006)	Vigor1	At my work, I felt bursting with energy.
		Vigor2	At my job, I felt strong and vigorous.
		Absorption1	Time flied when I was working.
		Absorption2	I felt happy when I was working intensely.
		Dedication1	I found the work that I did full of meaning and purpose.
		Dedication2	I was enthusiastic about my job.
	Job satisfaction (Schneider et al., 2003)	Job_sat1	Considering everything, how would you rate your overall satisfaction with the company at the present time?
		Job_sat2	I like the kind of work I do.
		Job_sat3	How satisfied are you with your involvement in the decisions that affect your work?
		Job_sat4	I have enough information to do my job well.
		Job_sat5	How do you rate this company in providing job security for people like yourself?
	P-O fit (Ng and Feldman, 2009)	Embed1	I fit with the company’s culture.
		Embed2	I feel like I am a good match for the company.
		Embed3	My values are compatible with the company values.
Control	Demographic	Seniority	Individual organizational tenure (years).
		Type of contract	Binary: 0-fixed term/other, 1-full time.
		Age	Individual age (years).
		Gender	Binary: 0-male, 1-female.

### 3.3 Data analysis

To identify and validate latent constructs from the survey data, we first employed Confirmatory Factor Analysis (CFA). CFA is a statistical technique commonly used in psychometric research to assess the validity and reliability of latent constructs (Brown, 2015). It evaluates the degree to which the observed data align with the theoretical framework by testing predefined relationships between observed variables and their underlying latent factors. Specifically, CFA provides insights into the number of latent constructs being measured and helps determine which items are associated with the same construct versus those that are linked to different constructs. This method relies on prior theory to specify the number of factors and the structure of factor loadings (Hair et al., 2019). CFA allows researchers to rigorously test measurement models, ensuring both construct validity and internal consistency.

Then, we used a Generalized Additive Model (GAM) to investigate the effect of the latent constructs emerging from CFA on Work-related stress.

GAM extend traditional linear regression by allowing non-linear relationships between predictors and the response variable through smooth functions (James et al., 2017; Wood, 2017). For a response variable $y_{i}$ $\left(i=1,\cdots ,n\right)$ , and p predictors $x_{ij}$ $\left(j=1,\cdots ,p\right)$ , the GAM framework can be expressed as Equation 1:


(1) $$y_{i}=\beta_{0}+\overset{p}{\underset{j=1}{\sum }}f_{j}\left(x_{ij}\right)+\varepsilon_{i}$$

where $\beta_{0}$ is the intercept, $f_{j}\left(x_{ij}\right)$ represents smooth functions capturing the non-linear effects of the predictors $x_{ij}$ , and $\varepsilon_{i}$ is the error term. The smooth functions $f_{j}\left(x_{ij}\right)$ are modeled as a weighted sum of basis functions:


(2) $$f_{j}\left(x_{ij}\right)=\overset{N_{j}}{\underset{z=1}{\sum }}\beta_{jz}b_{jz}\left(x_{ij}\right)$$

where $b_{jz}x_{ij}$ are basis functions, $\beta_{jz}$ are their corresponding coefficients, and $N_{j}$ is the number of basis functions for the j-th predictor. Various smoothing methods, such as cubic regression splines, thin-plate splines, and P-splines, can be used to model $f_{j}\left(x_{ij}\right)$ in Equation 2. The trade-off between smoothness and model fit is controlled by a smoothing parameter $\lambda_{j}$ specific to each smooth term, which penalizes the wiggliness of $f_{j}\left(x_{ij}\right)$ . Both the coefficients $\beta_{jz}$ and the smoothing parameters $\lambda_{j}$ are estimated using Restricted Maximum Likelihood (REML). This ensures that both the smooth terms and the linear predictors are estimated in a way that maximizes the restricted likelihood of the model.

All statistical analyses were conducted through the R software environment, using the ‘lavaan’ package for CFA (Rosseel, 2012) and the ‘mgvc’ package for GAM estimation (Wood, 2017).

## 4 Results

### 4.1 CFA and construct validity

Initially, we conduct CFA to assess the reliability and validity of the latent constructs (Fornell and Larcker, 1981). Table 3 presents the results of the measurement model, which were obtained using maximum likelihood estimation. The chi-square statistic was significant (*χ*^2^ = 448.824, d.f. = 215, *p* < 0.01), indicating a discrepancy between the expected model and the observed covariance matrix. However, given the small sample size and the sensitivity of the chi-square statistic to sample size (West et al., 2012, p. 211), we followed the guidelines proposed by Kline (2016) to assess model fit using alternative indices: Root Mean Square Error of Approximation (RMSEA), Comparative Fit Index (CFI), and Standardized Root Mean Square Residual (SRMR). The measurement model demonstrated a good fit, with indices either close to or exceeding their respective thresholds (RMSEA = 0.084; CFI = 0.939; SRMR = 0.064) (Byrne, 2016).

**Table 3:** CFA output.

Factor	Item	Std. loading	Cronbach’s alpha	Mean	SD
Work-life enrichment (CR = 0.800, AVE = 0.814)			0.853		
1	The things I did at work helped me deal with personal and practical issues at home.	0.772		3.52	1.58
2	The things I did at work made me a more interesting person at home.	0.817		4.32	1.58
3	The skills I used on my job were useful for things I had to do at home.	0.850		3.81	1.69
Work-life conflict (CR = 0.801, AVE = 0.851)			0.886		
1	Things I wanted to do at home did not get done because of the demands my job put on me.	0.892		2.97	1.59
2	My job produced strain that made it difficult to fulfil family duties.	0.793		2.42	1.47
3	The amount of time my job took up made it difficult to fulfil family responsibilities.	0.865		2.72	1.57
Engagement (CR = 0.891, AVE = 0.807)			0.916		
1	At my work, I felt bursting with energy.	0.852		5.03	1.39
2	At my job, I felt strong and vigorous.	0.828		4.84	1.44
3	Time flied when I was working.	0.732		5.35	1.27
4	I felt happy when I was working intensely.	0.653		4.94	1.33
5	I found the work that I did full of meaning and purpose.	0.849		4.83	1.45
6	I was enthusiastic about my job.	0.904		5.26	1.36
Job Satisfaction (CR = 0.836, AVE = 0.762)			0.866		
1	Considering everything, how would you rate your overall satisfaction with the company at the present time?	0.943		5.50	1.22
2	I like the kind of work I do.	0.762		5.57	1.20
3	How satisfied are you with your involvement in the decisions that affect your work?	0.764		4.96	1.46
4	I have enough information to do my job well.	0.704		5.18	1.26
5	How do you rate this company in providing job security for people like yourself?	0.596		5.81	1.22
P-O fit (CR = 0.895, AVE = 0.885)			0.917		
1	I fit with the company’s culture.	0.890		5.77	1.26
2	I feel like I am a good match for the company.	0.911		5.76	1.21
3	My values are compatible with the company values.	0.854		5.77	1.34
Work-related stress (CR = 0.724, AVE = 0.747)			0.754		
1	Did you feel capable of making decisions about the things that happened to you?	0.842		5.33	1.45
2	Were you able to concentrate on whatever you were doing?	0.532		5.26	1.16
3	Did you feel like you were playing a useful part in the things that happened to you?	0.826		5.51	1.14
Model fit statistics	$\chi^{2}$ = 448.824, d.f. =215, $\chi^{2}$ /d.f. = 2.08; CFI = 0.939, TLI = 0.920, RMSEA = 0.084; SRMR = 0.064				

CR, Composite reliability; AVE, Average variance extracted.

The factor and item loadings all exceeded 0.596, and the Average Variances Extracted (AVE) were above 0.75, providing strong evidence of convergent validity among our measures (Fornell and Larcker, 1981). Additionally, all measures demonstrated strong reliability, with composite reliabilities ranging from 0.724 to 0.895. The AVE for each construct exceeded the squared inter-factor correlations, indicating the distinctiveness of all four constructs (see Table 4). Overall, our constructs exhibit sound measurement properties (Fornell and Larcker, 1981; Hair et al., 2019).

**Table 4:** Construct correlations.

	Work-life enrichment	Work-life conflict	Engagement	Job satisfaction	P-O fit	Work-related stress
Work-life enrichment	**0.902**					
Work-life conflict	0.240	**0.922**				
Engagement	0.494	0.080	**0.898**			
Job satisfaction	0.526	0.108	0.850	**0.873**		
P-O fit	0.458	0.028	0.778	0.863	**0.941**	
Work-related stress	0.325	0.135	0.817	0.871	0.676	**0.864**

The diagonal values (in bold) represent the AVE, while the construct correlations are displayed below the diagonal.

Regarding the factor structure, a minimum of three items per factor were included, to provide adequate identification for the construct (Hair et al., 2019). In general, all Cronbach’s alpha coefficients exceeded 0.754, indicating good internal consistency (Cronbach, 1951).

### 4.2 Common method variance

Given that the data for this study was obtained from a single respondent within the same company, there is a potential risk of common method bias. To address this concern, we employed the Common Latent Factor (CLF) approach (Eichhorn, 2014; Podsakoff et al., 2012). This method involves introducing a new latent factor, representing common method variance, to which the observed items are loaded in addition to their respective theoretical constructs.

Furthermore, the loadings on the CLF were constrained to be equal across all items. The results of the Common Method Variance (CMV) test met the required criteria, with the overall shared variance explained by the CLF being 18.27%, which is significantly below the 50% threshold suggested by Eichhorn (2014).

### 4.3 Predictive analysis

**Figure 2:**
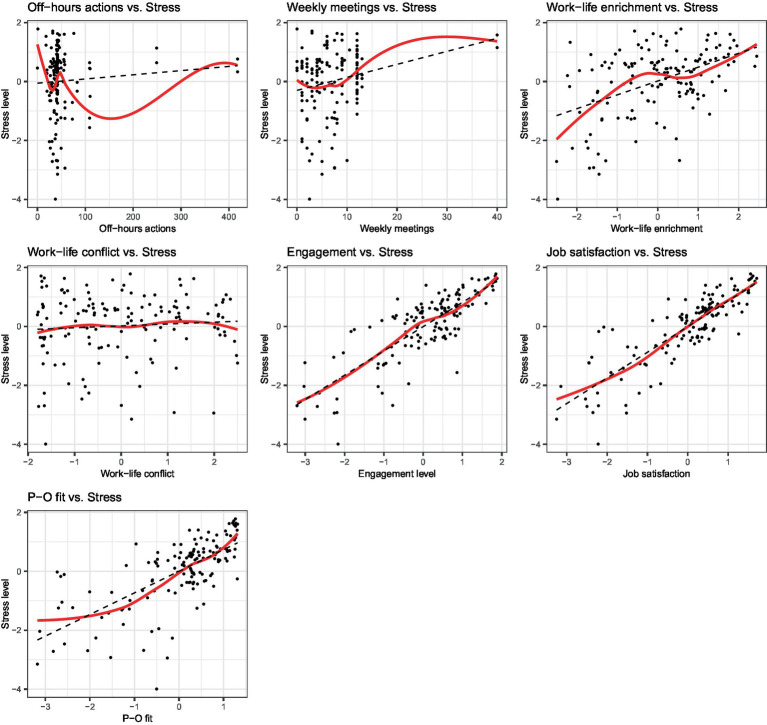
Non-linear relationships.

Following CFA, we evaluated the impact of each driver on work-related stress, incorporating additional control variables for a more comprehensive analysis. A graphical inspection of Figure 2, which shows the bivariate relationships between each independent variable and work-related stress, revealed the presence of nonlinear patterns. To address these potential nonlinear relationships, we employed a GAM specifying smooth terms (i.e., nonlinear functions) for variables exhibiting nonlinear associations with the dependent variable. In fact, unlike traditional linear models, which assume a fixed linear or parametric form for the relationship between the dependent variable and covariates, GAMs do not impose any *a priori* assumptions about the functional form of these relationships. This flexibility allows GAMs to effectively identify and estimate nonlinear effects of the covariates on the dependent variable. As shown in Figure 2, the two click-related variables (off-hours actions and weekly meetings), Person-organization fit and Work-life enrichment show nonlinear patterns in the relationship with Work-related stress. Thus, these variables were included as smooth terms in the model, which can be defined as follows:


(3) $$\begin{array}{l} \mathrm{Work}-{\mathrm{related stress}}_{i}=\beta_{0}+f_{1}\left(\mathrm{Off}-{\mathrm{hours actions}}_{i}\right)\\ +f_{2}\left({\mathrm{Weekly meetings}}_{i}\right)+f_{3}\left(\mathrm{Work}-{\mathrm{life enrichment}}_{i}\right)\\ +\beta_{1}\left(\mathrm{Work}-{\mathrm{life conflict}}_{i}\right)+\beta_{2}\left({\mathrm{Engagement}}_{i}\right)\\ +\beta_{3}\left(\mathrm{Job}\;{\mathrm{satisfaction}}_{i}\right)+f_{4}\left(\mathrm{Person}-\mathrm{organization}\;{\mathrm{fit}}_{i}\right) \end{array}$$

To test the appropriateness of the GAM, we tested it against a multiple linear regression model. Specifically, the ANOVA revealed a statistically significant reduction in residual deviance ( Δ Deviance = 4.63, *p* < 0.001) when transitioning from the linear model to the GAM, meaning that the latter captures non-linear relationships in the data more effectively than the linear model.

The Akaike Information Criterion (AIC) values, reported in Table 5, further support the superiority of the GAM (AIC_Model2_ = 115.457) over the linear model (AIC_Model1_ = 126.686), with the lower AIC reflecting a better trade-off between model complexity and goodness of fit. These findings underscore the importance of incorporating smooth terms for variables where non-linear effects are expected, as this enhances the model’s ability to represent the underlying data structure accurately.

**Table 5:** AIC comparison.

	df	AIC
Model 1 (Linear)	14.000	126.686
Model 2 (GAM)	31.711	115.457

In the Equation 3, both the parametric coefficients and the coefficients of the basis functions for the smooth terms were estimated simultaneously using REML, which jointly optimizes the parametric coefficients, the coefficients of the basis functions and the related smoothing parameter (*λ*). The parametric estimates from the regression model are presented in Table 6.

**Table 6:** Hierarchical GAMs.

Parametric coefficients						
	Model 1	Model 2	Model 3	Model 4	Model 5	Model 6
(Intercept)	0.000 (0.020)	0.121* (0.065)	0.326*** (0.120)	−0.062 (0.239)	−0.163 (0.238)	−0.057 (0.262)
Work–life conflict	0.238*** (0.029)	0.231*** (0.029)	0.227*** (0.029)	0.233*** (0.029)	0.203*** (0.025)	0.226*** (0.028)
Engagement	0.097 (0.071)	0.112 (0.071)	0.109 (0.070)	0.095 (0.070)	0.079 (0.061)	0.352*** (0.104)
Job satisfaction	−2.130*** (0.130)	−2.091*** (0.126)	−2.095*** (0.125)	−2.109*** (0.124)	−1.856*** (0.109)	−2.178*** (0.122)
Seniority		−0.050** (0.024)	−0.045** (0.023)	−0.048** (0.023)	−0.042** (0.020)	−0.033 (0.023)
Contract_1			−0.239** (0.115)	−0.257** (0.115)	−0.240** (0.102)	−0.297** (0.113)
Age				0.012* (0.007)	0.012** (0.006)	0.009 (0.007)
Gender_1					0.088 (0.092)	0.119 (0.101)
Engagement × Gender_1						−0.281*** (0.087)
R^2^ adjusted	0.867	0.908	0.901	0.908	0.91	0.918
n	144	144	144	144	144	144
AIC	91.41	90.39	88.68	87.52	86.72	77.19

Gender: 0–male, 1–female; Contract: 0–fixed term/other, 1–full time. ∗ *p* < 0.1; ∗∗ *p* < 0.05; ∗∗∗ *p* < 0.01.

**Figure 3:**
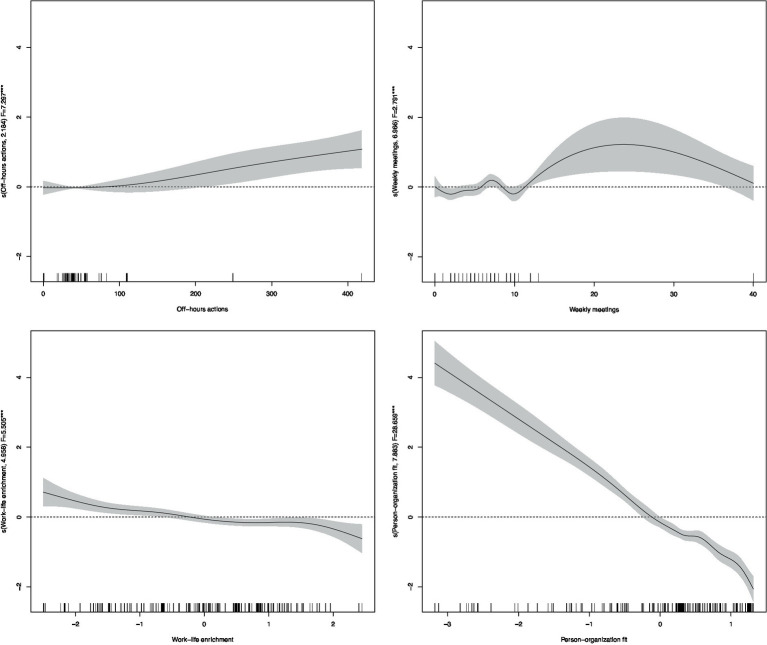
Smooth terms from GAM model.

For the smooth terms, we employed thin-plate regression splines, a type of penalized regression spline. Penalization mitigates overfitting by balancing the trade-off between model fit and smoothness, enhancing the generalizability of the results. Unlike traditional splines, thin-plate splines do not require predefined knot locations; instead, the basis functions are derived directly from the underlying data structure, ensuring that the smooth function is not overly influenced by arbitrary knot placement (Wood, 2003). The results for the smooth functions, including Effective Degrees of Freedom (EDF) and F-statistics, are reported in Table 7. Moreover, a graphical representation of the estimated smooth functions from the full model (Model 6) is provided in Figure 3, offering visual insights into the functional relationships between the predictors and work-related stress.

**Table 7:** Smooth terms.

	edf	F	edf	F	edf	F
	Model 1		Model 2		Model 3	
s(Off-hours actions)	2.313	3.994**	2.729	4.351***	2.161	5.443***
s(Weekly meetings)	7.189	1.408	7.357	1.790*	7.010	2.134**
s(Work–life enrich.)	5.446	3.889***	1.434	9.574***	1.721	9.837***
s(Person–Organization fit)	8.104	23.217***	8.059	23.795***	8.108	24.051***
	Model 4		Model 5		Model 6	
s(Off-hours actions)	2.261	4.865***	2.207	5.197***	2.184	7.297***
s(Weekly meetings)	6.960	1.946*	6.805	1.901*	6.966	2.791**
s(Work–life enrich.)	1.417	10.429***	1.532	9.352***	4.958	5.505***
s(Person–Organization fit)	8.290	24.344***	8.369	24.504***	7.884	28.659***

Models 1–6 refer to the different GAM specifications in Table 6. Asterisks denote statistical significance: *p < 0.1; **p < 0.05; ***p < 0.01.

The results of the hierarchical GAM provide several key insights into the factors influencing work-related stress in a digitalized work environment. Model 1 serves as the baseline specification, incorporating both the five constructs extracted from CFA and click metadata variables to evaluate their impact on work-related stress. Subsequent models (i.e., Models 2 through 6) sequentially introduce control variables, and the effects observed in the baseline model are consistently confirmed.

Regarding click metadata, the smooth functions in Figure 3 reveal that a significant amount of daily work performed outside regular hours, as well as prolonged time spent in virtual meetings, both contribute to increased work-related stress. These findings support Hypothesis 1 (H1), indicating that in a digitalized working context, the intensification and extension of work across time and space significantly increases work-related stress, particularly when associated with out-of-hours work and video calls. From Figure 3 we can also extract information regarding the amount of digital work that impact significantly on employees’ stress. In fact, an average of approximately 250 actions taken by a user during a working day outside regular hours significantly increases stress levels. Similarly, attending more than 11 meetings per week on average is associated with higher stress levels. These thresholds could serve as benchmarks for organizations to monitor and manage employees’ digital workloads effectively.

The results also strongly support Hypotheses 2a and 2b (H2a, H2b). Specifically, work-life enrichment is found to significantly reduce work-related stress (*F* = 3.889, *p* < 0.01), while work-life conflict significantly increases it. However, Hypothesis 3 (H3a, H3b) is not supported, as engagement does not exert a significant effect on work-related stress. This suggests that engagement may not be a critical factor in shaping stress levels within the digital work environment. The model provides strong evidence in favour of Hypothesis 4 (H4), demonstrating that higher levels of job satisfaction significantly reduce work-related stress (*p* < 0.01). This finding highlights the critical role of job satisfaction in mitigating stress in a digitalized workplace. Similarly, Hypothesis 5 (H5) is confirmed, as employees’ perceptions of person-organization (P-O) fit, or embeddedness, significantly decrease work-related stress (*F* = 23.217, *p* < 0.01).

Incorporating demographic variables (i.e., seniority, contract type, age, and gender) provided additional specificity to the findings. Model 2 highlights a modest but significant impact of organizational tenure on stress, with longer tenure associated with reduced stress, though this effect diminishes in later models. Model 3 shows that holding a full-time contract alleviates stress levels, while Model 4 identifies a positive relationship between age and stress, indicating higher stress levels among older employees. Finally, Model 6 incorporates an interaction between engagement and gender, revealing a significant moderating effect (p < 0.01). The negative coefficient suggests that engagement mitigates stress more effectively for females compared to their male counterparts.

Regarding model fit, the adjusted R^2^ values are notably high, ranging from 86.7% in Model 1 (AIC = 91.41) to 91.8% in Model 6 (AIC = 77.19), indicating an excellent fit for the estimated models. While GAMs are generally more effective with larger datasets, our sample size is sufficient for the model’s complexity. Model diagnostics, including high adjusted R^2^ values, decreasing AIC, indicate that the model complexity is adequately justified. The smooth term plots and associated confidence intervals exhibit well-behaved trends without excessive fluctuations, indicating that the smoothing penalties are effective in preventing overfitting. Finally, both parametric and smooth terms show statistically significant effects, providing strong evidence that the model’s estimates are stable and meaningful despite the sample size.

Additionally, during model fitting, we assessed concurvity, which arises when a smooth term or a combination of predictors is highly correlated with another smooth term or linear predictor (Hastie and Tibshirani, 1986). Concurvity can be considered the non-linear counterpart to collinearity and, if present, may result in unstable parameter estimates. In our analysis, as shown in Table 8, concurvity estimates remained below 0.50, indicating that it is not a concern in our model (Ramsay et al., 2003).

**Table 8:** Observed concurvity.

	Parameters	s(Off-hours actions)	s(Weekly meetings)	s(Person–Organization fit)	s(Work–life enrich.)
Parameters	1	0	0	0	0
s(Off-hours actions)	0	1	0.098	0.014	0.038
s(Weekly meetings)	0	0.108	1	0.14	0.11
s(Person–Organization fit)	0	0.139	0.061	1	0.275
s(Work–life enrich.)	0	0.043	0.052	0.315	1

## 5 Discussion

The redefinition of the boundaries between the material and the immaterial, the physical and the virtual, in terms of presence and remoteness, constitutes the hallmark of the incorporation of ICTs into organizational contexts (Schwarzmüller et al., 2018). Overall, the digital transformation has created new time and space dimensions of work, by erasing physical and time barriers (Bissola and Imperatori, 2018). Hence, the restructuring of an organization, involving people and technologies, can be understood as a people-driven organizational revolution triggered by the adoption of disruptive digital innovations (Nadkarni and Prügl, 2020).

As stated by Johnson et al. (2020, p. 405), technology has increasingly set the pace and method of work, enabling the workforce to access unlimited amounts of online information, to rapidly complete routine cognitive tasks, to deliver services in-person or remotely and to have dynamic collaborations with individuals or teams across different time zones around the world. On the other hand, the use of ICTs has produced constant connectivity, work fragmentation, heavier workload and a perpetual sense of urgency, also creating expectations that people need, or are obligated, to work faster and more efficiently (Ayyagari et al., 2011). In this regard, Tarafdar et al. (2019) identified techno-overload, techno-invasion, techno-uncertainty, techno-insecurity and techno-complexity as techno-stressors. Since the influence of technology on work-related stress also depends on employees’ perceptions of its effect on their job and work experience, as either an opportunity or a damage (Johnson et al., 2020; Tarafdar et al., 2019), this study has sought to explore the complexity of the relationship between the individual experience of work and the broader organizational process of digitalization.

As suggested by Qvarfordt and Lagrosen (2023), results from our empirical research demonstrate that digital technologies in the workplace have the potential to produce a wide range of effects and consequences on employees’ working experience.

More specifically, our findings show several significant interactions between digital activities reflecting the intensification and extension of working time–space (namely, off-hours actions and weekly meeting), employees’ attitudes towards their job and organization (namely, work-life interface, job satisfaction, person-organization fit), and work-related stress in a highly digitalized working context.

Firstly, we found that the digital time–space intensification and extension of working experience fostered by the digitalization of work and measured in terms of daily work performed outside regular office hours and extended time spent in virtual meetings significantly contributes to an increase in work-related stress. Although several studies emphasized the greater flexibility and control with respect to the time and place to work (Coenen and Kok, 2014; Ter Hoeven and Van Zoonen, 2015; Vega et al., 2015) and the possibility to create virtual teams (Cortellazzo et al., 2019), our results suggest that the ability to work from virtually any location at any time (Hassard and Morris, 2022), coupled with the requirement to be almost constantly available for an increasing number of virtual meetings that are both more frequent and longer in duration (Luebstorf et al., 2023) significantly impact stress levels among employees. Notably, we found that an average number of actions taken by the user in a working day outside office hours approaching 250 significantly increases employees’ stress, as does attending more than 11 meetings per week on average. These quantities could serve as early-warning indicators to proactively prevent critical stress levels. By integrating these indicators into organizational tools or dashboards, organizations can monitor employees’ digital activities and use these thresholds to identify and flag individuals who may be at higher risk of work-related stress. In this regard, it is recommended that organizational policies place greater emphasis on the risks associated with constant availability enabled by digital technology. To enhance employees’ well-being, it is necessary to limit work outside office hours, thereby promoting a complete switch-off from professional responsibilities and facilitating the restoration of physical and mental health. Moreover, the implementation of specific managerial practices is necessary to prevent the occurrence of Zoom fatigue. Measures such as promoting asynchronous communication, implementing policies like “no-email hours,” or enabling automatic delays for emails sent outside regular working hours can be effective. Furthermore, efforts should be made to both mitigate the stressors associated with virtual meetings and to ensure that videoconferences are scheduled with due consideration for their duration and frequency. Furthermore, our findings demonstrate that the use of digital technologies in the workplace is strongly related to the work-life interface dimension, as argued in Chesley (2014), thereby contributing to the extant literature which holds that work-life conflict produces stress-related outcomes (Anderson et al., 2002; Fiksenbaum, 2014), while work-life enrichment positively relates with individual’s mental health (Baral and Bhargava, 2010). In particular, the results reveal that perceived work-related stress increases significantly when the digitalization of work leads to greater conflict between life and work spheres (Stich et al., 2018). On the other hand, when work-life enrichment prevails, stress at work tends to decrease (Alieva and Powell, 2022). Moreover, the analysis indicates that higher levels of satisfaction with one’s own work, which can be positively affected by digitalization (Bolli and Pusterla, 2022), significantly reduce work-related stress, as also found by Ahsan et al. (2009) and Klassen et al. (2010). Additionally, the perception of person-organization fit appears to play a significant role in decreasing work-related stress, thus confirming the results reported by Mostafa (2016) and Chen et al. (2016). However, our study broadens the existing literature by demonstrating that the relationship among these employees’ attitudes (i.e., work-life interface, p-o fit and work-related stress) is significant in the new, contemporary working environment, and expanding the current understanding of the role assumed by employees’ subjective experiences and perceptions in the mitigation of stress in a working context deeply permeated by digital technologies.

These findings also have practical implications in terms of monitoring employees’ attitudes as factors influencing stress levels. Specifically, the formulation and submission of surveys to collect evidence of employees’ subjective perceptions of their work experience (in terms of job-satisfaction, p-o fit, engagement, work-life interface) would serve as a valuable resource in the development of strategies to mitigate work-related stress. By drawing upon the results of these surveys, organizations could imagine specific job design practices and HRM policies that are intended to enhance, for example, employees’ satisfaction with their job activities and responsibilities, as well as the alignment between individuals’ values and growth expectations, on the one hand, and the organizational culture and career opportunities, on the other.

Finally, although we initially supposed that job engagement would be a critical factor in influencing stress levels in a digitalized workplace (Hooi and Chan, 2023; Syrek et al., 2022;), this evidence does not emerge from our study, thus echoing Olsen et al. (2023) that found remote working to be unrelated to job engagement. However, as evidenced by the findings, engagement seems to mitigate the impact of gender on perceived work-related stress in a digitalized working context. Although previous research found that “there are no consistent results that indicate that women are less engaged than men or the opposite” (Banihani et al., 2013), since results showed no difference between the work engagement of men and women (Hartman and Barber, 2020),in our results female employees exhibited lower stress levels the more they were engaged. This reveals the complex relationship among work digitalization, gender, engagement and work-related stress, which should be further investigated in terms of organizational diversity and inclusion. Nevertheless, the current findings indicate that prospective gender-specific interventions designed to support female workers in the management of stress should incorporate strategies for enhancing engagement with their occupational activities.

Therefore, the primary contribution of this study is to provide new perspectives for interpreting the impact of digitalization on employees’ working experience, pointing out the role of employees’ attitudes in a digitalized workplace and showing how these subjective perceptions can influence stress levels also induced by the digitalization itself. Indeed, from the analysis of both the digital activities and the attitudes of employees, it appears that the dissolved spatial and temporal boundaries of work, that underlie the work-life conflict and are revealed in continuous video conferencing and out-of-hours work, lead to a significant increase in stress. Conversely, satisfaction with one’s job, beneficial management of the work-life interface (i.e., work-life enrichment) and the perception of fit with the organization were found to contribute to lower stress levels.

### 5.1 Limitations and further research

The findings of this study should be considered in light of its limitations. Firstly, the short collection period of Microsoft 365 click metadata (8 months) may not fully capture long-term effects of digitalization on stress. Secondly, the relatively small sample size and the focus on a single organization is problematic in terms of generalizing findings across different cultural contexts, industries and work environments. It would be certainly interesting to analyze a comprehensive record of digital activities over a longer period, which could provide additional insights into temporal patterns of stress. In this regard, further research with longitudinal data would enhance understanding of the ongoing impact of digitalization and its long-term stress implications for employees. Another important sample’s limitation consists in the significant male predominance that does not allow for an accurate investigation of the female component of the workforce and limits the ability to draw conclusion about gender-specific stressors and coping mechanism. To address gender differences more thoroughly, a more diverse sample should be included to extend this research and to allow for a more detailed discussion of the organizational dimensions of diversity and inclusion. Moreover, beyond click metadata, which represents digital activities, this study examines digital transformation in terms of a highly digitalized work environment. This is a fairly limited perspective on the various dimensions of organizational processes of digitalization and leaves room for further research, which could be complemented by other measures of employees’ digital interactions. Additionally, although the Likert-scale self-report measures are standard, they may introduce biases such as social desirability or response fatigue. Therefore, further qualitative data and interviews could enable a more in-depth exploration of the reasons behind certain attitudes toward digitalization and stress.

Indeed, as workplaces’ digitalization is becoming more and more prominent, employees are constantly required to interact with an increasing range of digital assets within the organizational ecosystem (Malik et al., 2023). In this regard, “AI is widely heralded as a new and revolutionary general purpose technology that will transform the world of work” (Charlwood and Guenole, 2022). Therefore, further academic research into AI is now particularly recommended to explore employees’ attitudes towards AI, with the aim of understanding how they perceive and interact with this technology in the workplace. Finally, it would provide useful insights to examine both the digital work activities and attitudinal variables included in this research in relation to the employees’ physical health or, for example, to the highly contemporary phenomenon of quiet quitting (Mahand and Caldwell, 2023).

## 6 Conclusion

In conclusion, this research effectively highlights the complexities of digitalization and its dual impact on employees’ well-being, specifically providing a comprehensive exploration of the interplay between digitalization and work-related stress. The present study contributes to the extant academic literature by revealing the influence of both subjective perceptions of employees and digital activities on work-related stress in a digitalized workplace. The results indicate that employees’ attitudes towards their job and organization have the potential to reduce stress levels also induced by the time–space intensification and extension of working experience within deeply digitalized work settings.

Potential contributions to advance theoretical understanding and practical implications are suggested to both researchers and practitioners, especially about work-life balance and stress management in digital environments.

## Data Availability

The dataset for this manuscript is not publicly available because of privacy and ethical restrictions (it contains information that could compromise research participants’ privacy/consent). Requests to access the dataset should be directed to Tommaso Fabbri (tommaso.fabbri@unimore.it), PI of the research project “Work Datafication and Behavioral Visibility in the Digital Workplace” which our empirical study is part of.
